# Overexpression of modified human TRβ1 suppresses the growth of hepatocarcinoma SK-hep1 cells in vitro and in xenograft models

**DOI:** 10.1007/s11010-018-3357-1

**Published:** 2018-04-20

**Authors:** Xiaoxiang Peng, Yuntao Zhou, Yanli Sun, Wei Song, Xiangying Meng, Chunling Zhao, Ronglan Zhao

**Affiliations:** 1 Department of Laboratory Medicine, Weifang Medical University, Weifang, 261053 Shandong China; 2 Key Discipline of Clinical Laboratory Medicine of Shandong Province, Affiliated Hospital of Weifang Medical University, Weifang, 261053 Shandong China; 3 Central Hospital of Zibo, Zibo, 255020 Shandong China; 4 Key Laboratory of Biological Medicine in Universities of Shandong Province, Weifang Medical University, Weifang, 261053 Shandong China

**Keywords:** TRβ1, Tumor suppressor, Hepatocarcinoma, SK-hep1 cells

## Abstract

Association studies suggest that TRβ1 functions as a tumor suppressor. Thyroid hormone receptors (TRs) mediate transcriptional responses through a highly conserved DNA-binding domain (DBD). We previously constructed an artificially modified human TRβ1 (m-TRβ1) via the introduction of a 108-bp exon sequence into the corresponding position of the wild-type human TRβ1 (TRβ1) DBD. Studies confirmed that m-TRβ1 was functional and could inhibit the proliferation of breast cancer MDA-MB-468 cells in vitro. To understand the role of m-TRβ1 in liver tumor development, we adopted a gain-of-function approach by stably expressing TRβ (m-TRβ1 and TRβ1) genes in a human hepatocarcinoma cell line, SK-hep1 (without endogenous TRβ), and then evaluated the effects of the expressed TRβ on cancer cell proliferation, migration, and tumor growth in cell-based studies and xenograft models. In the presence of 3,5,3-l-triiodothyronine (T3), the expression of TRβ in SK-hep1 cells inhibited cancer cell proliferation and impeded tumor cell migration through the up-regulation of *4-1BB, Caspase-3,* and *Bak* gene expression; down-regulation of *Bcl-2* gene expression; and activation of the Caspase-3 protein. TRβ expression in SK-hep1 led to less tumor growth in xenograft models. Additionally, the anti-tumor effect of m-TRβ1 was stronger than that of TRβ1. These data indicate that m-TRβ1 can act as a tumor suppressor in hepatocarcinoma and its role was significantly better than that of TRβ1.

## Introduction

TRs are members of a superfamily that comprises the nuclear receptors and ligand-dependent transcription factors that mediate the biological actions of thyroid hormone (T3) in differentiation, growth, development, and the maintenance of metabolic homeostasis [1, 2]. TRs are encoded by two human genes, namely, *THRA* and *THRB*; furthermore, four DNA- and T3-binding isoforms, namely, TRα1, TRβ1, TRβ2, and TRβ3 (only present in rat), are generated by alternative mRNA splicing and differential promoter usage [2]. These isoforms are functional receptors and share a common structure in several regions ranging from the N-terminus to the C-terminus: an N-terminal activation domain (A/B), a conserved DNA-binding domain (DBD) (C), a hinge domain (D), and a ligand-binding domain (E/F). TRs regulate target gene transcription by interacting with specific DNA sequences, known as thyroid hormone response elements (TREs) [3]. Several recent studies have strongly suggested that TRβ plays an important role in cell proliferation and malignant transformation [4–7]. Low or even no expression of TRs and alterations in TR genes, especially TRβ1, have been identified in many types of cancer [8–12]. Studies have also demonstrated that mutations in TRβ1 are closely associated with many cancers [13–16]. Many studies have confirmed that TRβ1 plays as a tumor suppressor in human cancers [17–19].

We previously identified a novel rat TRβ (rTRβ) isoform with an extra-large DBD, named TR beta delta (TRβΔ, GenBank number: DQ191165). rTRβΔ is highly homologous to rat TRβ1 (rTRβ1) and contains an additional 108 nucleotides or 36 amino acids in the DBD, which alters this highly conserved domain [20]. Nevertheless, studies have demonstrated that rTRβΔ is a functional TR, that rTRβΔ has transcription factor characteristics, and that rTRβΔ exhibits a greater tumor-suppressive ability in vitro than rTRβ1. Subsequently, we constructed an artificial *m-TRβ1* by introducing this new 108-bp exon into the DBD of human *TRβ1*. m-TRβ1 was functional in MDA-MB-468 cells and inhibited MDA-MB-468 cell proliferation by promoting apoptosis in the presence of T3. However, the roles of m-TRβ1 in other cancer cell lines in vitro and in tumorigenesis in vivo are currently unknown.

In the present study, we adopted a gain-of-function approach by expressing the human TRβ (m-TRβ1 and TRβ1) gene in human hepatocarcinoma cells, namely, SK-hep1. We stably expressed the TRβ gene in SK-hep1 cells and evaluated the effects of TRβ expression on cell proliferation, apoptosis, migration, and tumor development in xenograft models. We found that TRβ gene expression in the presence of T3 promoted SK-hep1 apoptosis, inhibited SK-hep1 proliferation, and impeded migration. These inhibitory responses were mediated by the up-regulation of *4-1BB, Caspase-3*, and *Bak* gene expression, down-regulation of *Bcl-2* gene expression, and activation of the Caspase-3 protein due to the expression of TRβ. Moreover, the expression of TRβ in SK-hep1 significantly reduced SK-hep1 tumor growth in xenograft models. Further analysis indicated that the effects of m-TRβ1 were stronger than those of TRβ1. Thus, m-hTRβ1 could act as a tumor suppressor in hepatocarcinoma cells.

## Materials and methods

### Animals and reagents

A human hepatocarcinoma cell line (SK-hep1) was obtained from the Cell Bank of the Chinese Academy of Sciences (Shanghai, China). 293T cells and lentiviral vector GV358 were purchased from GeneChem (Shanghai, China). DMEM was purchased from Gibco (CA, USA). The Annexin V-FITC apoptosis detection kit, the NE-PER™ nuclear and cytoplasmic extraction reagents, and thyroid hormone receptor beta-1 antibody were purchased from (Thermo Fisher, MA, USA). Other reagents were obtained as follows: GAPDH antibody (Santa Cruz, CA, USA); the Bcl-2, 4-1BB, Bak, Histone H3, and active Caspase-3 antibodies (Bioss, Beijing, China); the TRIzol total RNA extraction reagent, the In-Fusion™ PCR cloning kit, and quantitative real-time PCR detection kit (Takara, Dalian, China); M-MLV reverse transcriptase (Invitrogen, CA, USA); KOD-Plus-Ver polymerase (TOYOBO, Tokyo, Japan); the Caspase-3 spectrophotometric assay kit (NANJING KEYGEN BIOTECH. CO., LTD, Nanjing, China); and MTT (3-(4,5-dimethylthiazol-2-yl)-2,5-diphenyltetrazolium bromide) (Promega, Beijing, China). Four-week-old female BALB/c nude mice (15–18 g) were obtained from Shanghai Lingchang BioTech CO., Ltd (Shanghai, China). Protocols involving animals used in this study were approved by the Institutional Animal Care and Use Committee of Weifang Medical University.

### In vitro experiments

#### Construction of GV358-*TRβ1 and* GV358-*m-TRβ1* vectors

Using PCR, we obtained the total sequence of wild-type human *TRβ1* (*TRβ1*) and artificially modified the human TRβ1 (m-TRβ1) cDNA from pcDNA3.1-*wt-hTRβ1* and pcDNA3.1-*m-hTRβ1* (previously constructed and stored by our team). The forward primer was 5′-GAGGATCCCCGGGTACCGGTCGCCACCATGACTCCCAAC AGTATGACAG-3′, and the reverse primer was 5′-TCCTTGTAGTCCATACCATCCTCGAACACTTCCAAGAAC-3′. The PCR product was directionally cloned into the lentiviral vector GV358, which was linearized with *Age* I with the In-Fusion™ PCR cloning kit according to the manufacturer’s protocol. The constructed expression vectors, namely, GV358-*TRβ1* and GV358-*m-TRβ1*, were confirmed via sequence analysis.

### Viral packaging

The GV358-*TRβ1*, GV358-*m-TRβ1*, or GV358 empty vector plasmid was co-transfected with the Helper 1.0 and Helper 2.0 packaging helper plasmids (GeneChem, Shanghai, China) into 293T cells with a mass ratio of 4:3:2. The culture supernatants were harvested at 48 h post-transfection to purify the lentiviruses expressing TRβ1, m-TRβ1, or the control. The lentiviruses were purified via ultracentrifugation (Beckman, CA, USA) at 25,000 rpm for 2 h at 4 °C, and the viral titers were measured with endpoint dilution by ELISA (the HIV-1 p24 Antigen ELISA 2.0 assay kit according to the manufacturer’s instructions). SK-hep1 cells were infected with concentrated viruses (GV358-*TRβ1*, GV358-*m-TRβ1*, or GV358 empty vector) at a multiplicity of infection of 10, and the infection solution was discarded 12 h after infection. Because the lentivirus vector contained GFP, the infection efficiency was determined by observing GFP-positive cells under a fluorescence microscope 72 h after infection. At 72 h post-infection, the SK-hep1 cells that stably expressed TRβ1 (SK-hep1-*TRβ1*) and m-TRβ1 (SK-hep1-*m-TRβ1*) were used for subsequent experiments. SK-hep1 cells were infected with the empty vector GV358 and served as control cells (SK-hep1-*Neo*). A portion of these cells was collected for Western blot. The total protein was resolved by 10% SDS–PAGE and transferred from the gel to PVDF membranes. The PVDF membranes were blocked for non-specific reactivity with TBS-T containing 5% non-fat milk for 2 h at room temperature (RT) and then incubated with primary antibodies (TRβ1 or H3, 1:1000 dilution) at 4 °C overnight. TRβ1 recognizes an epitope in the A/B domain of TRβ1, namely, amino acid residues 1-101, which comprises a common sequence between TRβ1 and m-TRβ1. After thoroughly washes in TBS-T, the membranes were incubated with the secondary HRP antibodies (anti-mouse, 1:5000 dilution) for 1.5 h at RT. After thorough washes with TBS-T, the membranes were visualized using enhanced chemiluminescence and Kodak films (Kodak, Rochester, USA).

### Cell proliferation and apoptosis assays

MTT assays were employed to measure cell proliferation. SK-hep1-*TRβ1* cells, SK-hep1-*m-TRβ1* cells, and SK-hep1-*Neo* cells were seeded at a density of 1 × 10^4^/mL into 96-well plates, and then, 10 nM T3 was added to the intervention groups. After 48 h, a sterile-filtered MTT solution (20 µL, 5 mg/mL) was added to each well, followed by incubation for 4 h at 37 °C. Then, the formazan crystals were solubilized in dimethyl sulfoxide. The absorbance at 570 nm was recorded using a microplate reader (BIO-RAD, CA, USA), and the background absorbance at 630 nm was subtracted.

SK-hep1-*TRβ1* cells, SK-hep1-*m-TRβ1* cells, and SK-hep1-*Neo* cells were seeded in 12-well plates, and then, 10 nM T3 was added to the intervention groups. After 48 h of culture, cells were harvested and stained with FITC-conjugated Annexin V and propidium iodide for 10 min at RT and detected by flow cytometry (BD, New Jersey, USA).

### Wound healing assay

SK-hep1-*TRβ1* cells, SK-hep1-*m-TRβ1* cells, and SK-hep1-*Neo* cells were seeded at 1 × 10^6^ cells per well in six-well plates. A pipette tip was used to introduce wounds to confluent cells, plates were washed with PBS, and culture medium (without serum) was added. Cells were further cultured in the medium with or without T3 (10 nM). At regular intervals, a camera system with an inverted microscope was used to visualized cell migration at ×100 magnification. The migration rate was quantified by measuring the distances between the edges of wound, and the percentage of migration was determined as the ratio of the migrated distance to the initial distance of the wound [21].

### Real-time fluorescent quantitative PCR (RT-qPCR) and Western blot

SK-hep1-*TRβ1* cells, SK-hep1-*m-TRβ1* cells, and SK-hep1-*Neo* cells were seeded in 6-well plates, and then, 10 nM T3 was added to the intervention groups. After 48 h, the cells were harvested for total RNA and protein extractions. Total RNA was extracted using the TRIzol reagent. mRNA (2 µg) was reverse transcribed into total cDNA in a 20 µL reaction mixture, and the mRNA levels of *4-1BB, Bcl-2, Bak,* and *Caspase-3* were analyzed by RT-qPCR, using the *Gapdh* gene as a reference gene. PCR reactions were performed in iQ5TM (BIO-RAD, USA) and detected with SYBR Green. The primers for each gene are shown in Table 1. The PCR cycling conditions were as follows: 95 °C for 30 s, followed by 35 cycles of 95 °C for 5 s, 56 °C for 20 s, and 72 °C for 20 s. The CT values of all genes from the different samples were gathered, and the raw data were calibrated to the data for *Gapdh*. The mRNA of each sample was normalized, and the relative expression level of each gene was represented as ${2^{ - \Delta \Delta {C_{\text{T}}}}}.$

**Table 1:** Primers for each gene

Gene name	Gene symbol	Accession number	Amplicon length (bp)	Primer sequences
Glyceraldehyde-3-phosphate dehydrogenase	GAPDH	NM_001256799.2	162	Forward: gCATCCTgggCTACACTgAg Reverse: CCACCACCCTgTTgCTgTAg
B-cell lymphoma-2	Bcl-2	NM_000633.2	178	Forward: TgTgTgTggAgAgCgTCAAC Reverse: gACAgCCAggAgAAATCAAAC
TNF receptor superfamily member 9	4-1BB	NM_001561.5	164	Forward: gTTgCTCTTCCTgCTgTTC Reverse: ATCCTCCTTCTTCTTCTTCTg
Caspase 3	Caspase-3	NM_004346.3	162	Forward: ggAAgCgAATCAATggACTC Reverse: TTCCCTgAggTTTgCTgC
BCL2 antagonist/killer 1	Bak	NM_001188.3	155	Forward: TACCgCCATCAgCAggAAC Reverse: TCTgAgTCATAgCgTCggTTg

Western blot analysis was carried out as described above. The primary antibodies used were against TRβ1 (1:1000), GAPDH (1:1000), Bcl-2 (1:100), active Caspase-3 (1:200), and Bak (1:100).

### Caspase-3 activity assay

SK-hep1-*TRβ1* cells, SK-hep1-*m-TRβ1* cells, and SK-hep1-*Neo* cells were seeded in 6-well plates, and then, 10 nM T3 was added to the intervention groups. After 48 h, the cells were lysed in RIPA buffer, and the total protein concentration of each lysate was determined using the BCA protein assay kit from PIERCE (PIERCE, USA). Caspase-3 activity was measured using the Caspase-3 spectrophotometric assay kit according to manufacturer’s instructions.

### In vivo experiments

#### BALB/c nude mouse transplantation

Four-week-old female BALB/c nude mice were purchased from Shanghai Lingchang BioTech CO., Ltd [Certificate of Quality No: SCXK (Shanghai) 2013-0018]. Mice were housed four per cage and provided with free access to water, food, and bedding at all times. The mice were randomly separated into three groups comprising ten mice: (1) the SK-hep1-*TRβ1* cell group, (2) the SK-hep1-*m-TRβ1* cell group, and (3) the SK-hep1-*Neo* cell group. SK-hep1-*TRβ1* cells, SK-hep1-*m-TRβ1* cells, and SK-hep1-*Neo* cells were digested and respectively implanted into the skin of the right forelimb armpit of each nude mouse in a total volume of 200 µL (4 × 10^6^) cells resuspended in D-Hanks without fetal bovine serum (FBS), under aseptic conditions. Two weeks after cell inoculation, primary tumor outgrowth was monitored three times per week by measuring the tumor width (*W*) and length (*L*) with a digital caliper. The tumor volume was calculated as follows: π/6 × *L* × *W* × *W*. Tumor growth was evaluated up to day 56 after cell inoculation. Eight weeks after inoculation, the tumor-bearing mice were anesthetized with Peltobalrbitalum Natricum, imaged using the Small Animal Live Imaging System (Berthold Technologies, Germany), and sacrificed by cervical dislocation. The tumors were removed, weighed, and used to examine the parameters described below.

#### Western blot and immunohistochemical analysis

Two samples were randomly selected from each group to extract total protein for the detection of TRβ1 and m-TRβ1 protein. The Western blot analysis was carried out as described above. The tumor tissue was fixed in 10% buffered formalin for 24 h and then embedded in paraffin. Four-micrometer sagittal sections were made and processed for immunohistochemistry according to a standard protocol. Primary antibodies used were against active Caspase-3 (dilution 1:200), 4-1BB (1:50 dilution), Bcl-2 (1:200 dilution), and Bak (1:200 dilution). Staining was developed with 3,3-diaminobenzidine (DAB). Cells that were positive for active Caspase-3, 4-1BB, Bcl-2, or Bak were counted for quantitative analysis.

### Statistical analysis

All measurement data are expressed as the mean ± SD and were analyzed with SPSS 17.0. The single-factor analysis of variance was used for the multigroup comparisons. Each result was compared using the Student–Newman–Keuls test. A value of *p* < 0.05 was considered significant.

## Results

### Generation of *wt-TRβ1* and *m-TRβ1* lentiviral expressing vectors

**Fig. 1:**
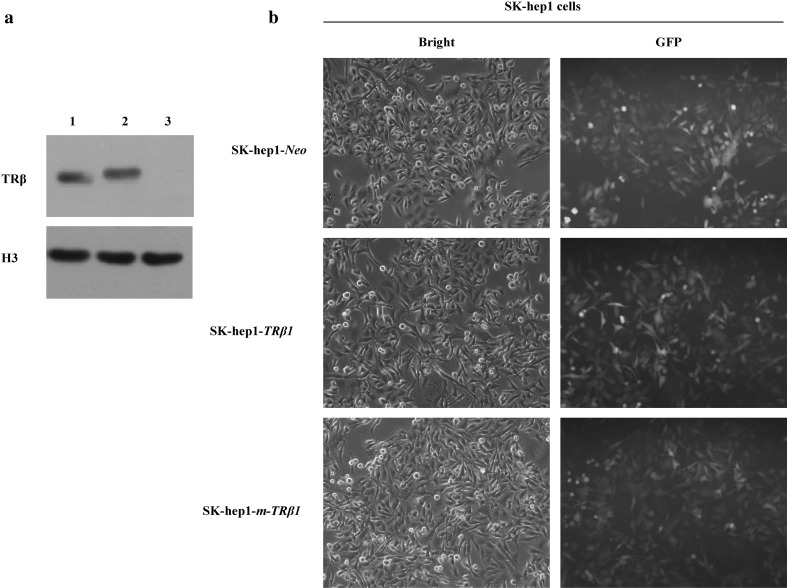
Establishment of human hepatocarcinoma SK-hep1 cell lines stably expressing TRβ1 and m-TRβ1. **a** TRβ1 and m-TRβ1 were expressed in SK-hep1-TRβ cells (lanes 1 and 2) but not in control SK-hep1-*Neo* cells (lane 3). Western blot analysis was carried out as described in Materials and methods. **b** Representative micrographs of SK-hep1 cells under a ×100 magnification microscope and under an inverted fluorescence microscope at 72 h following lentiviral infection (GV358-*TRβ1*, GV358-*m-TRβ1*, or GV358 empty vector)

A full-length coding sequence of *TRβ1* (1386 bp) or *m-TRβ1*(1494 bp) was directionally cloned into the GV358 lentiviral vector to obtain the GV358-*TRβ1* expression plasmid. This sequence was completely consistent with the sequence published in GenBank and with the GV358-*m-TRβ1* expression plasmid. The sizes of the TRβ1 and m-TRβ1 proteins were approximately 52 and 57 kD, respectively, as predicted from the aa sequence, and the proteins were expressed in SK-hep1 cells (without endogenous TRβ1 and m-hTRβ1) (Fig. 1a). GFP was used as a reporter gene. GV358 (empty vector, *TRβ1* or *m-TRβ1*) was successfully transduced into SK-hep1 cells since more than 80% of the cells in each group were GFP-positive by fluorescence microscopy at 72 h post-infection (Fig. 1b).

### m-TRβ1 inhibits the growth and migration of SK-hep1 cells

**Fig. 2:**
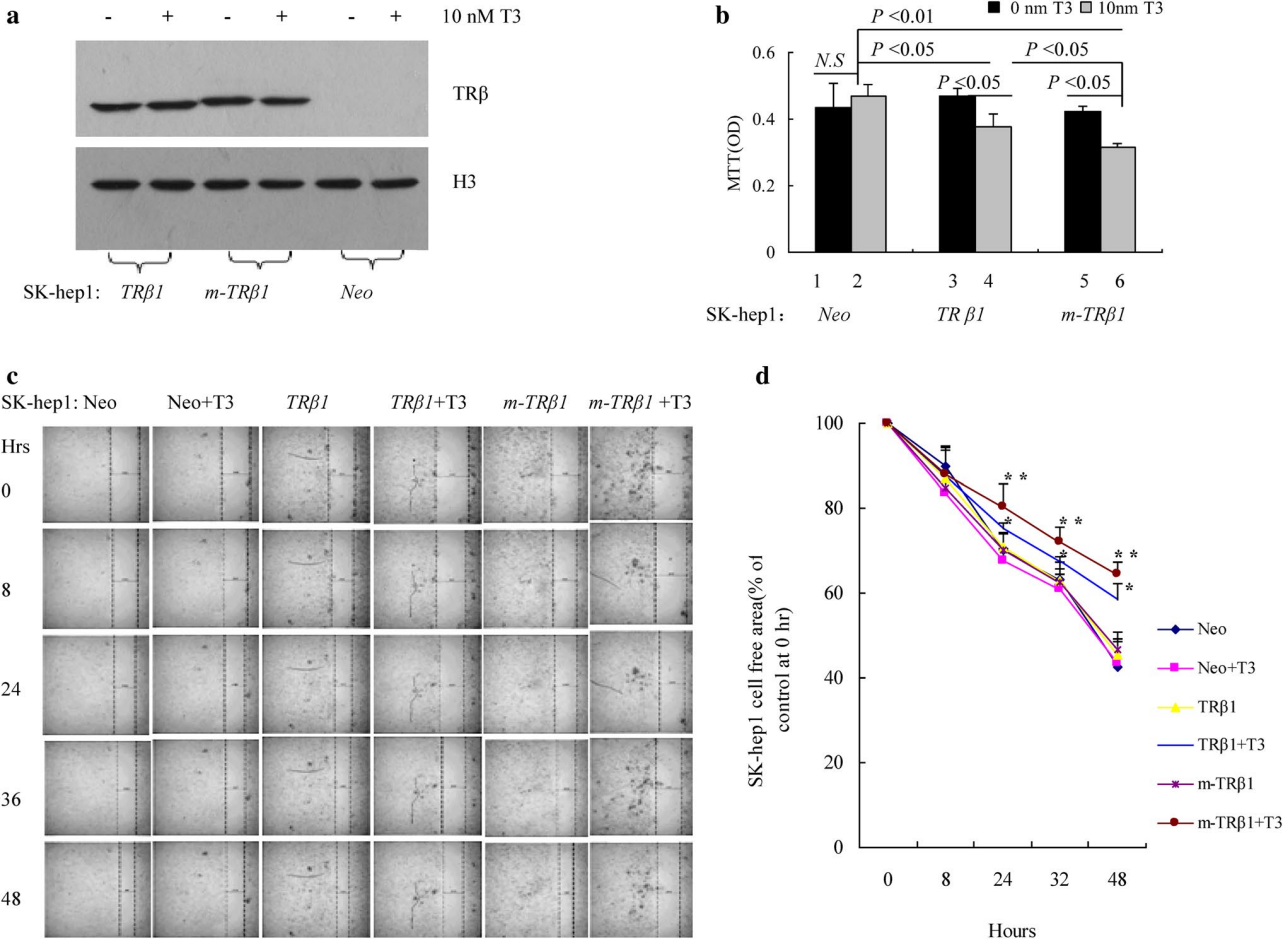
Stable expression of TRβ in SK-hep1 cells reduces cell growth and migration. **a** Western blot analysis of the expression of TRβ1 and m-TRβ1 in SK-hep1-*TRβ1* cells, SK-hep1-*m-TRβ1* cells, and SK-hep1-*Neo* cells. Western blot analysis was carried out as described in Materials and methods. **b** Cell growth was analyzed with MTT assays as described in Materials and Methods. Data are presented as the mean ± SD (*n* = 3), and the *p* values are shown. **c** Representative cell pictures of wound healing in SK-hep1-*TRβ1* cells, SK-hep1-*m-TRβ1* cells, and in control SK-hep1-*Neo* cells at 0, 8, 24, 36, and 48 h (Hrs) in the presence or absence of T3 as marked. **d** Cell migration rates determined from results shown in **c**. T3 inhibited the migration rates of the TRβ-expressing SK-hep1 cells (TRβ1 and m-TRβ1) relative to the rate of the control SK-hep1 cells (*Neo*). The effect of m-TRβ1 was stronger than that of TRβ1 on inhibiting cell migration in the presence of 10 nM T3. Data are presented as the mean ± SD. **p < 0.01, SK-hep1-*m-TRβ1* cells in the presence of T3 versus SK-hep1-*m-TRβ1* cells in the absence of T3, Neo control cells (presence or absence of T3) or SK-hep1-*TRβ1* cells in the absence of T3. *p < 0.05, SK-hep1-*TRβ1* cells in the presence of T3 versus SK-hep1-*TRβ1* cells in the absence of T3, Neo control cells (presence or absence of T3) or SK-hep1-*m-TRβ1* cells (presence or absence of T3)

To elucidate the functional consequences of the re-expression of m-TRβ1 in SK-hep1 cells, we generated SK-hep1 cells that stably expressed TRβ (TRβ1, m-TRβ1) or control SK-hep1 cells that stably expressed the empty vector GV358 (Neo). The protein abundance of TRβ in the SK-hep1*-TRβ1* cells and SK-hep1*-m-TRβ1* cells was confirmed by Western blot analysis (Fig. 2a), whereas no TRβ was detected in the control SK-hep1-*Neo* cells (Fig. 2a). When the cells were cultured in medium containing 10 nM T3 without FBS, cancer cell proliferation was clearly lower for SK-hep1-*m-TRβ1* cells than for SK-hep1-*TRβ1* cells and SK-hep1-*Neo* cells (*p* < 0.05) (Fig. 2b). Significant differences were also observed between SK-hep1-*TRβ1* cells and SK-hep1-*Neo* cells (*p* < 0.05) (Fig. 2b), whereas no T3 effect was observed on the proliferation of the SK-hep1-*Neo* cells that did not express TRβ.

As shown in Fig. 2c, d, T3 had no apparent effect on the cell migration of SK-hep1-*Neo* cells. Interestingly, in the absence of T3, the migration rates of the SK-hep1-*m-TRβ1* and SK-hep1-*TRβ1* cells were similar to that of the SK-hep1-*Neo* cells. However, in the presence of T3, the migration rates of the SK-hep1-*m-TRβ1* cells and SK-hep1-*TRβ1* cells were significantly slower than those of the SK-hep1-*TRβ1* cells and SK-hep1-*m-TRβ1* cells without T3. In the presence of 10 nM T3, the inhibition of cell migration by m-TRβ1 was stronger than that by TRβ1 (Fig. 2c–d). Quantitative analysis showed that migration was significantly inhibited in both groups (*p* < 0.05). Data are presented as the mean ± SD, and the *p* values are shown.

### m-hTRβ1 promotes the apoptosis of SK-hep1 cells

**Fig. 3:**
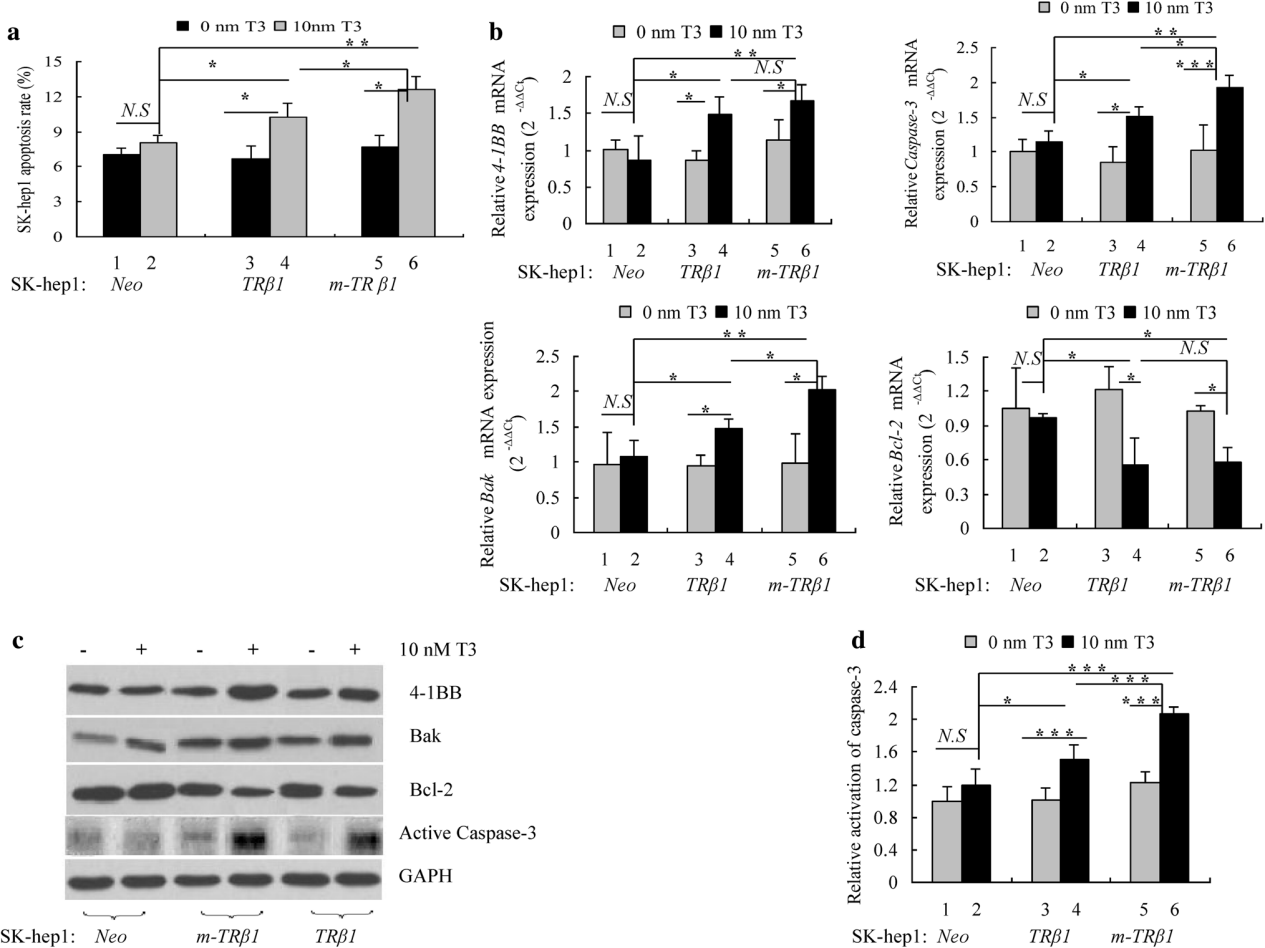
Stable expression of TRβ in SK-hep1 cells promotes cell apoptosis. SK-hep1 cell apoptosis was detected with Annexin V-FITC. **a** The total number of apoptotic cells was determined by calculating the sum of the early apoptotic cells (Annexin V-FITC^+^/PI^−^) and the late apoptotic cells (Annexin V-FITC^+^/PI^+^) detected by flow cytometry, and the data are presented as the mean ± SD. and the *p* values are shown (*p < 0.05, **p < 0.01). **b** TRβ regulates the expression of genes involved in cell apoptosis. The relative expression levels of the transcripts for apoptosis-related genes were estimated by RT-qPCR in the SK-hep1-*TRβ1*, SK-hep1-*m-TRβ1* and SK-hep1-*Neo* cells. Data were normalized to the amount of *Gapdh* mRNA and are represented as ${2^{ - \Delta \Delta {C_{\text{T}}}}}.$ Data are presented as the mean ± SD, and the *p* values are shown (*p < 0.05, **p < 0.01, ***p < 0.001). **c** Western blot analysis of apoptosis-related proteins in SK-hep1 cells. **d** Colorimetric assay of Caspase-3 activation. Caspase-3 activity was estimated by spectrophotometry. Data are presented as the mean ± SD, and the *p* values are shown (*p < 0.05, ***p < 0.001)

To understand how m-hTRβ1 expression inhibited SK-hep1 cell growth and migration in vitro, the effects of m-TRβ1 on SK-hep1 cell apoptosis were measured. SK-hep1-*m-TRβ1* cells, SK-hep1-*TRβ1* cells, and SK-hep1-*Neo* cells were cultured with or without T3. Flow cytometric analysis showed that cancer cell apoptosis was clearly higher among the SK-hep1-*m-TRβ1* cells than among the SK-hep1-*TRβ1* cells and SK-hep1-*Neo* cells (*p* < 0.05) (Fig. 3a). Significant differences were also observed between the SK-hep1-*TRβ*1 cells and SK-hep1-*Neo* cells (*p* < 0.05); however, no T3 effect on the apoptosis of the Neo cells was observed (Fig. 3a). These flow cytometric analysis results prompted us to evaluate how the expression of m-TRβ1 promoted the apoptosis of SK-hep1. Therefore, the levels of apoptosis-related genes were estimated by RT-qPCR. The RT-qPCR results showed that compared with the control Neo cells, in the presence of T3, m-hTRβ1 and TRβ1 caused a drastic increase in the *4-1BB, Bak*, and *Caspase-3* mRNA levels and a reduction in the *Bcl-2* mRNA level in SK-hep1 cells (*p* < 0.05); moreover, the effects of m-hTRβ1 were stronger than those of TRβ1 in the presence of 10 nM T3 (*p* < 0.05) (Fig. 3b). The Western blot results also showed that the expression levels of the 4-1BB, Bak, active Caspase-3, and Bcl-2 proteins (Fig. 3c) were consistent with the corresponding mRNA levels described above. Caspase-3 activity was also verified in cultured SK-hep1 cells using the Caspase-3 spectrophotometric assay kit. Compared with the control SK-hep1-*Neo* cells, in the presence of T3, the m-hTRβ1 and TRβ1 cells showed a gradual but significant up-regulation in Caspase 3 activity (*p* < 0.05) (Fig. 3d).

### m-TRβ1 expression inhibits SK-hep1 cell tumor growth in mouse xenograft models

**Fig. 4:**
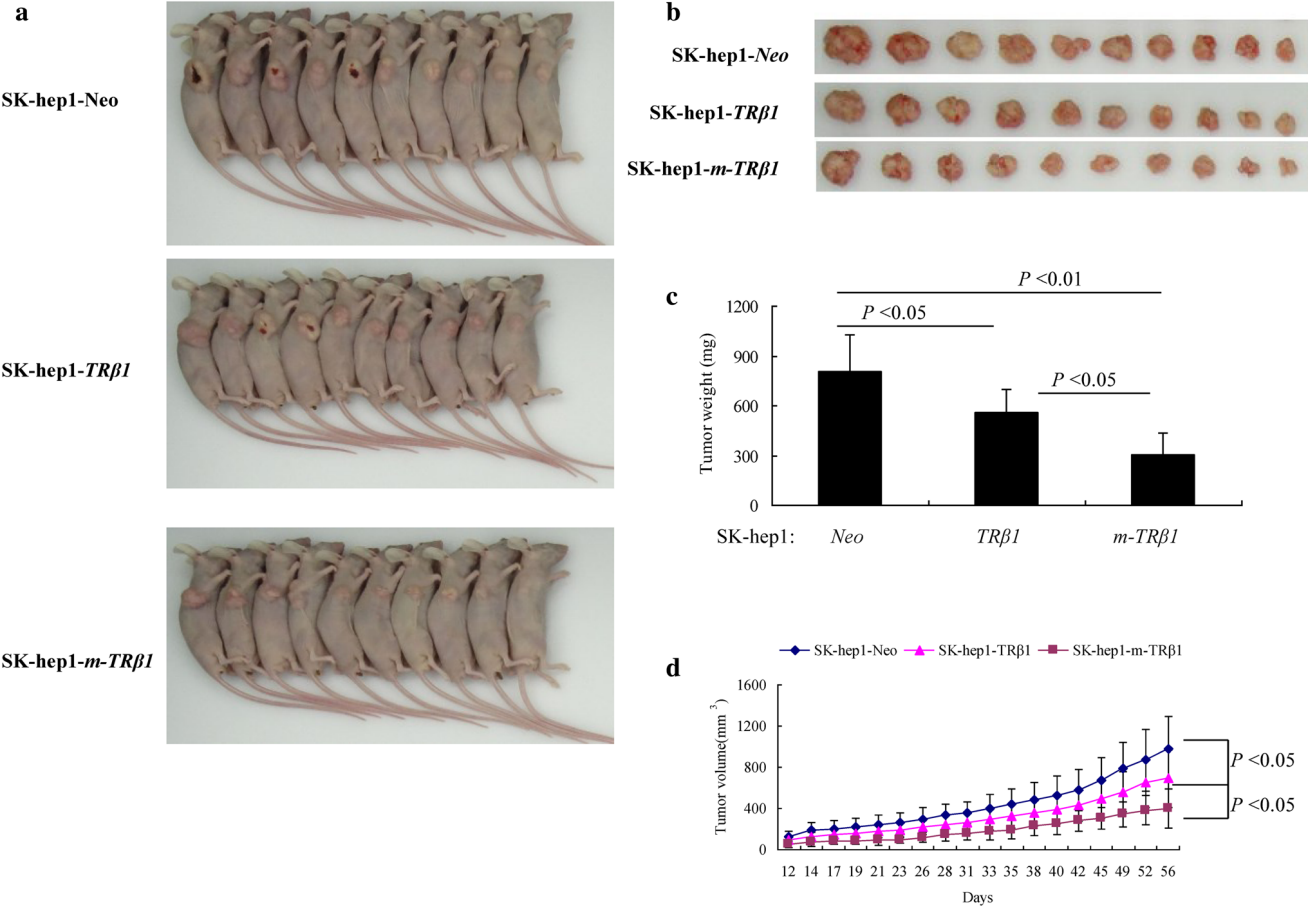
Comparison of the growth rates of the tumors derived from the SK-hep1-*TRβ1*, SK-hep1-*m-TR*β1, SK-hep1-*Neo* cells. **a** Representative pictures of the tumor-bearing mice. **b** Dissected tumors. **c** Tumors were dissected at the endpoint, and their weights were determined. Data are presented as the mean ± SD, and the *p* values are shown. **d** Beginning 2 weeks after cell inoculation, the tumor sizes were measured three times per week, and the rates of tumor growth were compared. The data are expressed as the mean ± SD (*n* = 10), and the *p* values are shown

To ascertain the role of m-TRβ1 in SK-hep1 cells, we inoculated SK-hep1-*m-TRβ1* cells, SK-hep1-*TRβ1* cells, and SK-hep1-*Neo* cells into nude mice. A nude mouse xenograft model of human hepatocarcinoma was established, and the tumor formation rate was 100%. As shown in Fig. 4a, b, the size of the tumor derived from the SK-hep1-*m-TRβ1* cells was clearly smaller than that of the tumor derived from the SK-hep1-*TRβ1* cells, and the size of the tumor derived from the SK-hep1-*TRβ1* cells was clearly smaller than that of the tumor derived from the control Neo cells. As demonstrated by the quantitative comparison shown in Fig. 4c, the tumor weights of the SK-hep1-*m-TRβ1* and SK-hep1-*TRβ1* tumors were significantly lower than those of the SK-hep1-*Neo* tumors (*p* < 0.01 and *p* < 0.05, respectively). As shown in Fig. 4d, the tumor growth rates of the SK-hep1-*m-TRβ1* and SK-hep1-*TRβ1* tumors were markedly slower than the growth rate of the control SK-hep1-*Neo* tumors. Among the three groups, the tumor growth rate of the SK-hep1-*m-TRβ1* tumor group was lowest.

**Fig. 5:**
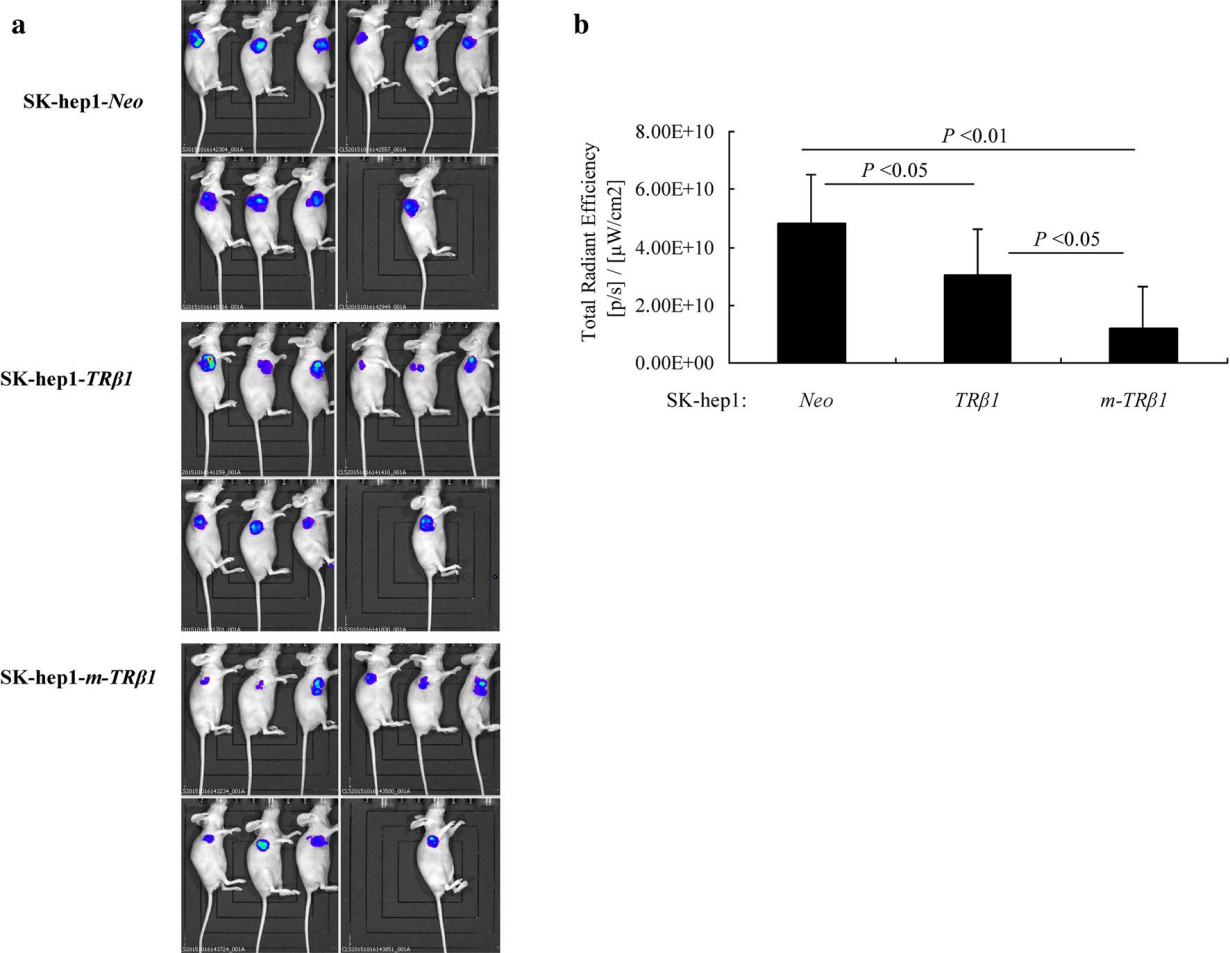
Observation of in vivo biofluorescence imaging of the SK-hep1-GFP tumors in the three groups BALB/c nude mice. **a** GFP expression in the inoculated SK-hep1 cells (stably expressing TRβ1, m-TRβ1, or Neo) observed with an in vivo biofluorescence imaging system. The total amount of fluorescence in the area indirectly reflected the number of fluorescently labeled cells or the size of the tumor volume. **b** The total radiation efficiency of each group. The data are expressed as the mean ± SD (*n* = 10), and the *p* values are shown

SK-hep1-*m-TRβ1* cells, SK-hep1-*TRβ1* cells, and SK-hep1-*Neo* cells inoculated into the nude mice expressed GFP, so GFP expression was observed with the in vivo biofluorescence imaging system. The total amount of fluorescence in the area was used to indirectly reflect the number of fluorescently labeled cells or the size of the tumor volume. All nude mice were euthanized on the 56th day, and the mean fluorescence intensity of GFP was determined using the small animal live imaging system. The mean fluorescence intensity of the SK-hep1-*m-TRβ1* cells group was the lowest among the three groups (*p* < 0.05) (Fig. 5a, b). Consistent with the cell-based findings described above, the in vivo results indicated that m-TRβ1 could act as a tumor suppressor in SK-hep1 cells and that the effects of m-TRβ1 were significantly stronger than those of TRβ1.

**Fig. 6:**
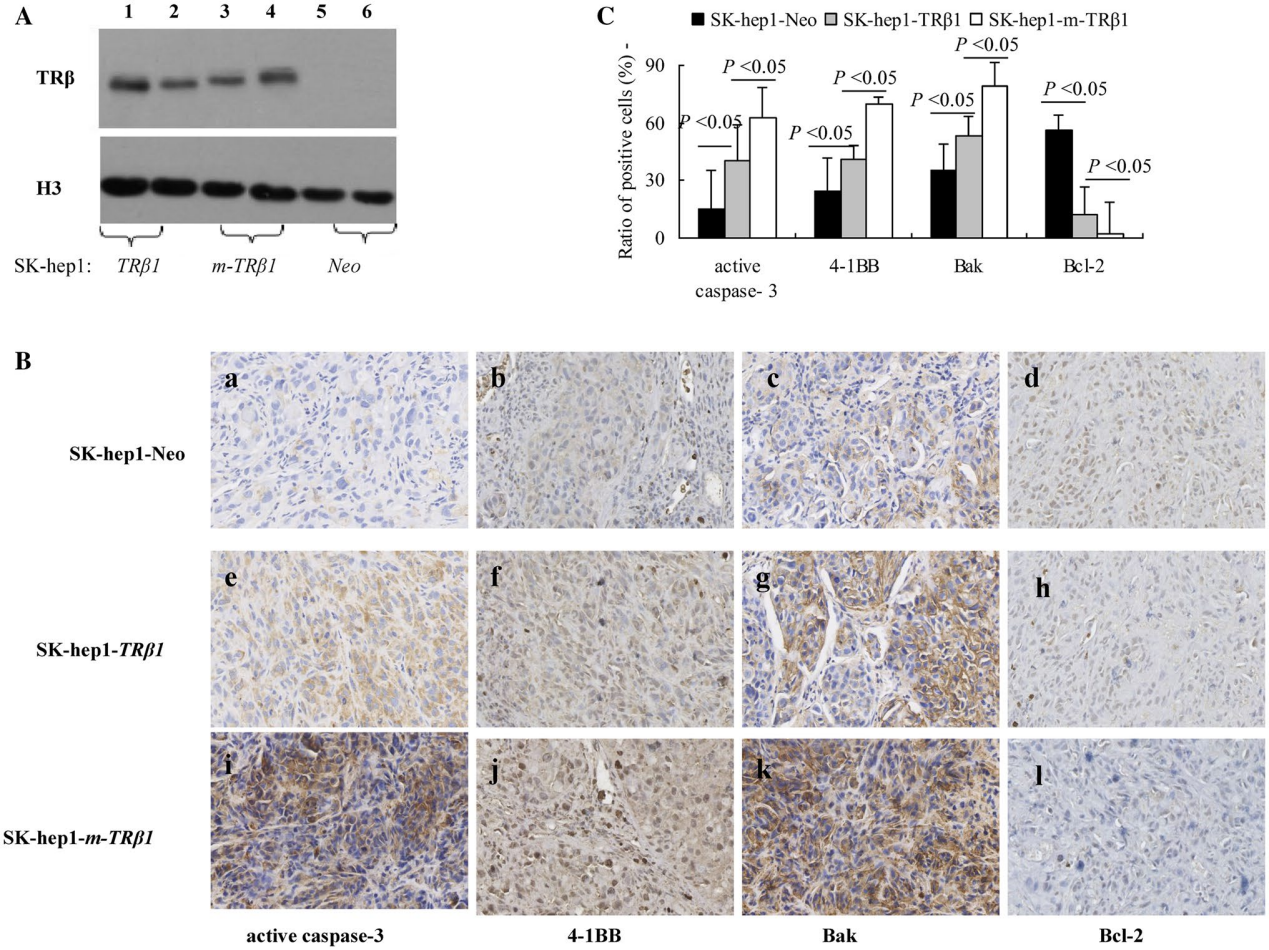
Western blot and immunohistochemical analysis in tumor tissues. **a** Western blot analysis of TRβ1 and m-TRβ1 in tumors. Tumors were excised from the injection sites (right forelimb armpits) of nude mice, and Western blot analysis was carried as described in Materials and methods. TRβ1 (lanes 1 and 2) and m-TRβ1 (lanes 3 and 4) were expressed in the tumor tissues (SK-hep1-*TRβ1* group and SK-hep1-*m-TRβ1* group) but not in the SK-hep1-*Neo* control group (lanes 5 and 6). **b** Immunohistochemical staining for active Caspase-3, 4-1BB, Bcl-2, and Bak (200×). Sections of the tumors derived from the Neo control cells (panels a–d), SK-hep1-*TRβ1* cells (panels e–h), and SK-hep1-*m-TRβ1* cells (panels i–l) were treated with anti-active Caspase-3 antibodies (panels a, e and i), anti-4-1BB antibodies (panels b, f and j), anti-Bak antibodies (panels c, g and k), or with anti-Bcl-2 antibodies (panels d, h, and l) as described in Materials and methods. **c** These positive cells were counted from three different sections and expressed as the percentage of positive cells versus the total number of cells examined. The data are expressed as the mean ± SD (*n* = 10). The *p* values are shown

We assessed whether the slow growth of the SK-hep1-*m-TRβ1* tumors resulted from increased apoptosis. First, we confirmed that the TRβ1 and m-TRβ1 proteins were expressed in the SK-hep1-*TRβ1* and SK-hep1-*m-TRβ1* tumors but not in the SK-hep1-*Neo* control tumors (Fig. 6a). Second, apoptosis-related proteins were confirmed by immunohistochemical analysis of active Caspase-3, 4-1BB, Bak, and Bcl-2 as markers in the tumors derived from the SK-hep1-*TRβ1* cells, SK-hep1-*m-TRβ1* cells, and SK-hep1-*Neo* cells. Only a few cells within the SK-hep1-*Neo* tumors were stained with the anti-active Caspase-3, 4-1BB and Bak antibodies (Fig. 6b, panel a–c). However, more cells within the SK-hep1-*TRβ1* tumors than within the SK-hep1-*Neo* tumors were stained with the anti-active Caspase-3, 4-1BB, and Bak antibodies (Fig. 6b, panel e–g), and most cells within the SK-hep1-*m-TRβ1* tumors were intensely stained with the anti-active Caspase-3, 4-1BB, and Bak antibodies (Fig. 6b, panel i–k). More cells in the SK-hep1-*Neo* tumors (Fig. 6b, panel d) than in the SK-hep1-*TRβ1* (Fig. 6b, panel h) and SK-hep1-*m-TRβ1* tumors (Fig. 6b, panel l) were stained positively for Bcl-2. Among the three groups, the positive staining rate in the SK-hep1-*m-TRβ1* tumors was lowest. The number of positively stained cells was counted, and the quantitative data are shown in Fig. 6c. Taken together, these data indicated that more tumor cells were underwent apoptosis in the SK-hep1-*m-TRβ1* tumors and that increased apoptotic activity in the m-TRβ1 tumors contributed to decreased tumor growth.

## Discussion

Previous studies that have shown a close association between the reduced expression and high mutation rate of the *TRβ1* gene and several human cancers support the hypothesis that TRβ1 can act as a tumor suppressor [8–13]. TRβ1 was previously shown to suppress tumor invasiveness and metastasis in human liver hepatocarcinoma cells (TR-deficient). When TRβ1 was re-expressed in these cells, cell proliferation and migration were inhibited in vitro, and tumor growth was suppressed in xenograft models [21]. We previously constructed an artificially modified human TRβ1 (m-hTRβ1) via the introduction of an extra 108-bp exon sequence into the corresponding position of the wild-type human TRβ1 (TRβ1) DBD. Studies have shown that m-TRβ1 can function as a tumor suppressor in a human breast cancer cell line, MDA-MB-468, in vitro. At present, the detailed effects and mechanisms of m-TRβ1 in human hepatocarcinoma cells are unclear.

In the present study, we further showed that m-TRβ1 could also act as a tumor suppressor in another cancer cell line, namely, the SK-hep1 human hepatocarcinoma cell line. We adopted a gain-of-function approach by expressing TRβ (m-TRβ1 and TRβ1) in SK-hep1 cells. In vitro, the re-expression of TRβ in SK-hep1 cells increased apoptosis, decreased cell proliferation, and impeded cancer cell migration. Moreover, in addition to these cell-based results, we showed that the growth of tumors derived from TRβ-expressing SK-hep1 cells in xenograft models was inhibited in vivo. The abovementioned effects of m-TRβ1 were significantly stronger than those of TRβ1. Studies have shown that TRβ1 can inhibit the proliferation and migration of hepatocarcinoma cells and other cancer cells [2, 4, 6, 15, 23–25]. Cell apoptosis is a complicated biological process that is associated with complex signaling pathway responses. The activation of cysteine proteases, particularly caspases, is a key intracellular regulator of cell apoptosis, and Caspase-3 is an important mediator of apoptosis [26]. TRβ expression induces apoptosis in breast cancer cells via an increase in cleaved PARP and Caspase-3 levels [27–29]. One study has shown that thyroid hormone induces the expression of 4-1BB, which encodes a tumor necrosis factor (TNF) receptor superfamily protein, and the activation of caspases in a thyroid hormone receptor-dependent manner [30]. *Bak* is a gene that promotes tumor cell apoptosis and belongs to the *Bcl-2* gene family. Bak can promote apoptosis by inhibiting the anti-apoptotic activity of Bcl-2 and Bcl-xl or by activating a pathway that is similar to that of the TNF death receptors. The involvement of pro-apoptotic Bak in the course and treatment of hepatocarcinoma has been reported [31, 32]. The level of Bak is reduced or even non-detectable in hepatocarcinoma cells, and this reduction in Bak contributes to the development and progression of hepatocarcinoma [33, 34]. In the present study, we found that in the presence of T3, m-TRβ1 inhibited proliferation and migration and promoted the apoptosis of SK-hep1 cells by up-regulating *Caspase-3, 4-1BB*, and *Bak* expression, increasing Caspase-3 activity and down-regulating *Bcl-2* expression (see Figs. 3, 6). The present study also demonstrates that the expression of m-TRβ1 impedes SK-hep1 tumor development in vivo (see Figs. 4, 5). At the same time, the abovementioned effects of m-TRβ1 are obviously stronger than those of TRβ1. These results suggest that modification of the DBD region may alter the TRβ activation intensity of target genes. Thus, the present study provides evidence to support the idea that m-TRβ1 can act as a tumor suppressor in human hepatocarcinoma development and progression. Other potential mechanisms that may underlie the m-TRβ1-mediated anti-proliferative effect await further studies in the future.

Our study demonstrates that m-TRβ1 expression in SK-hep1 cells decreases cancer cell proliferation, impedes tumor cell migration, and inhibits tumor growth in vivo by up-regulating *Caspase-3, 4-1BB,* and *Bak* expression, increasing Caspase-3 activity, and down-regulating *Bcl-2* expression. In addition, the anti-tumor effects of m-TRβ1 are significantly stronger than those of TRβ1. These findings indicate that m-TRβ can act as a tumor suppressor in hepatocarcinoma.
